# Protein–Protein Interactions Essentials: Key Concepts to Building and Analyzing Interactome Networks

**DOI:** 10.1371/journal.pcbi.1000807

**Published:** 2010-06-24

**Authors:** Javier De Las Rivas, Celia Fontanillo

**Affiliations:** Bioinformatics & Functional Genomics Research Group, Cancer Research Center (CiC-IBMCC, CSIC/USAL), Salamanca, Spain; Whitehead Institute, United States of America

This *PLoS Computational Biology* tutorial was presented at ISMB 2009

Decades of research into cell biology, molecular biology, biochemistry, structural biology, and biophysics have produced a remarkable compendium of knowledge on the function and molecular properties of individual proteins. This knowledge is well recorded and manually curated into major protein databases like UniProt [1], [2]. However, proteins rarely act alone. Many times they team up into “molecular machines” and have intricate physicochemical dynamic connections to undertake biological functions at both cellular and systems levels. A critical step towards unraveling the complex molecular relationships in living systems is the mapping of protein-to-protein physical “interactions”. The complete map of protein interactions that can occur in a living organism is called the interactome [3]. Interactome mapping has become one of the main scopes of current biological research, similar to the way “genome” projects were a driving force of molecular biology 20 years ago.

Efficient large-scale technologies that measure proteome-wide physical connections between protein pairs are essential for accomplishing a comprehensive knowledge of the protein interactomes. In recent years, given an explosive development of high-throughput experimental technologies, the number of reported protein–protein interactions (PPIs) has increased substantially. Large collections of PPIs produce “omic” scale views of protein partners and protein memberships in complexes and assemblies [4]. Over the same period as the development of large-scale technologies, efficient collection of a lot of small-scale experimental data published in relevant scientific journals is also taking place. This data compilation work is just as essential to achieving comprehensive knowledge of the interactome. Important efforts have been made to build public repositories that integrate information from large- and small-scale PPI experiments reported in the scientific literature. A compendium of PPI databases can be found in http://www.pathguide.org/.

To achieve appropriate understanding of PPIs and to design better ways for analyzing and interpreting them, this educational review presents several essential concepts and definitions intended to facilitate the use of PPI information both by computational and experimental biologists.

The report is divided into five sections and a summary: (a) *PPI definition*; a definition of a protein-to-protein interaction compared to other biomolecular relationships or associations. (b) *PPI determination by two alternative approaches: binary and co-complex*; a description of the PPIs determined by the two main types of experimental technologies. (c) *The main databases and repositories that include PPIs*; a description and comparison of the main databases and repositories that include PPIs, indicating the type of data that they collect with a special distinction between experimental and predicted data. (d) *Analysis of coverage and ways to improve PPI reliability*; a comparative study of the current coverage on PPIs and presentation of some strategies to improve the reliability of PPI data. (e) *Networks derived from PPIs compared to canonical pathways*; a practical example that compares the characteristics and information provided by a canonical pathway and the PPI network built for the same proteins. Last, a short summary and guidance for learning more is provided.

## PPI Definition

The first step needed is to define precisely what protein–protein interactions are. Commonly they are understood as physical contacts with molecular docking between proteins that occur in a cell or in a living organism in vivo. As discussed previously [5], [6], the issue of whether two proteins share a “functional contact” is quite distinct from the question of whether the same two proteins interact directly with each other. Any protein in the ribosome or in the basal transcriptional apparatus shares a functional contact with the other proteins in the complex, but certainly not all the proteins in the particular complex interact. Indubitably, the existence of many other types of functional links between biomolecular entities (genes, proteins, metabolites, etc.) in living organisms should not be confused with protein physical interactions. Investigating these functional links requires different experimental techniques designed to find such specific types of relationships, for example, double mutant synthetic lethality to find genetic interactions [7] or transcriptome expression profiling to find gene co-expression [8]. Identification of other types of protein interactions (protein–DNA, protein–RNA, protein–cofactor, or protein–ligand) is also important for a comprehensive study of the interactome, but again these types of data should not be mixed or confused with PPI data.

The physical contact considered in PPIs should be specific, not just all proteins that bump into each other by chance. It also should exclude interactions that a protein experiences when it is being made, folded, quality checked, or degraded. For example, all proteins at one point “touch” the ribosome, many touch chaperones, and most make contact with the degradation machinery. In many experimental assays, such generic interactions are rightfully filtered out. Therefore, the definition of PPI has to consider (1st) the interaction interface should be intentional and not accidental, i.e., the result of specific selected biomolecular events/forces; and (2nd) the interaction interface should be non-generic, i.e., evolved for a specific purpose distinct from totally generic functions such as protein production, degradation, and others.

That PPIs imply physical contact between proteins does not mean that such contacts are static or permanent. The cell machinery undergoes continuous turnover and reassembly. Some protein assemblies are stable because they constitute macromolecular protein complexes and cellular machines, for example ATP synthase (eight different proteins in mammals) or cytochrome oxidase (13 proteins in mammals). These proteins included in complexes are called “subunits”. Other protein assemblies are only built to carry out transient actions, for example, the activation of gene expression by the binding of transcription factors and activators on the DNA promoter region of a gene.

Another essential element for defining PPIs is the biological context. Not all possible interactions will occur in any cell at any time. Instead, interactions depend on cell type, cell cycle phase and state, developmental stage, environmental conditions, protein modifications (e.g., phosphorylation), presence of cofactors, and presence of other binding partners.

## PPI Determination by Two Alternative Approaches: Binary and Co-Complex

**Figure 1:**
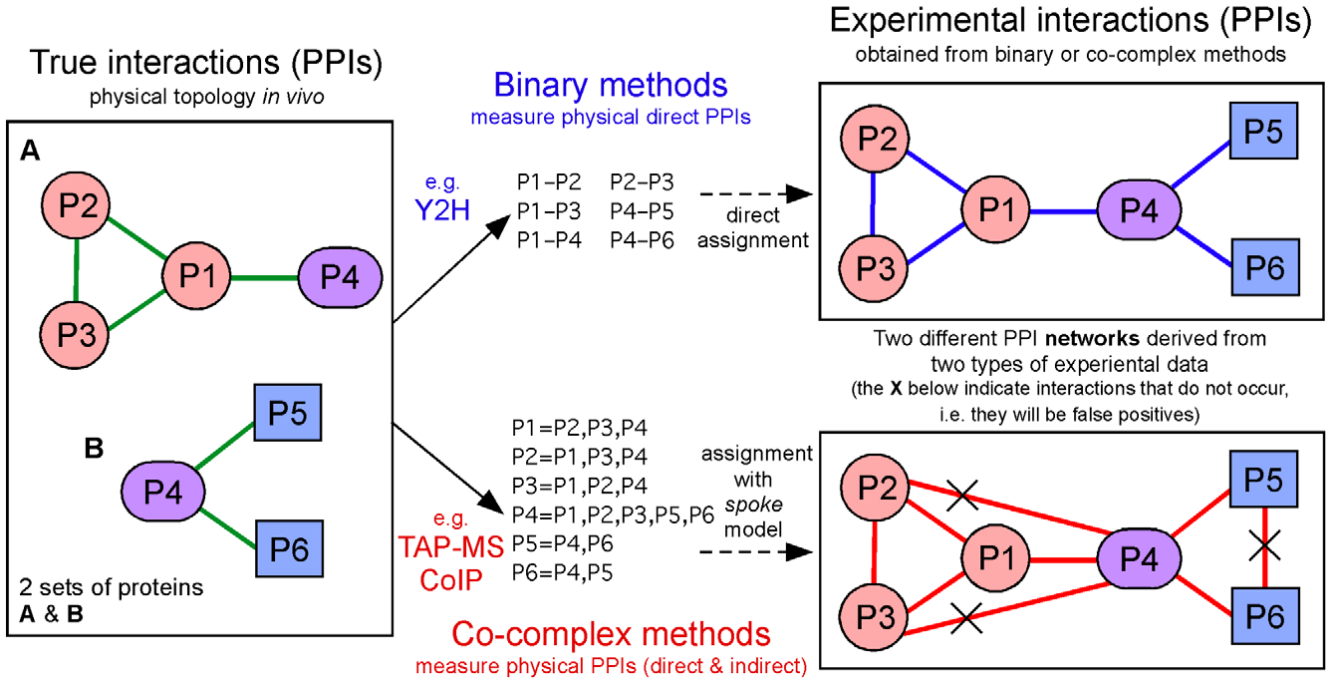
Binary methods and co-complex methods: two approaches to determine PPIs. The two most widely used experimental proteomic techniques applied to measure PPIs are yeast two-hybrid (Y2H) and tandem affinity purification coupled to mass spectrometry (TAP-MS); the former technique is a binary method (which measures physical direct interactions between protein pairs), and the latter a co-complex method (which measures physical interactions between groups of proteins without distinguishing whether they are direct or indirect). The interactions shown in the left panel (green links) correspond to the true interactions existing between two groups of proteins (set **A** with four proteins and set **B** with three proteins). The interactions shown in the right panels correspond to the networks derived from the experimentally measured interactions existing between the six proteins analyzed: the network in the top right panel (blue links) presents the interactions obtained using a binary method; the network in the bottom right panel (red links) presents the interactions obtained using a co-complex method. The red links are calculated applying the spoke model to the TAP-MS experimental data, but three of the interactions deduced (links with an X) do not occur.

Experimental determinations of interactions between proteins are done at either a large or small scale with two main technologies that produce different types of PPI data. The techniques that measure direct physical interactions between protein pairs are “binary” methods, while the techniques that measure physical interactions among groups of proteins, without pairwise determination of protein partners, are “co-complex” methods [9]. The most often used binary and co-complex methodologies are, respectively, yeast two-hybrid (Y2H) [10] and tandem affinity purification coupled to mass spectrometry (TAP-MS) [11]. Both are widely applied in large-scale investigations. Co-complex methods measure both direct and indirect interactions between proteins. The most common approach is based on the pre-selection of one protein tagged with a molecular marker (the bait protein), which is used to catch or “fish out” a group of proteins (prey proteins) followed by a biochemical technique to “pull-down” and separate them from a mix. In this way, what takes place is a co-purification of protein groups. Another common co-complex approach, based on protein antibody recognition, is co-inmunoprecipitation (CoIP) [5]. The experimental results obtained with co-complex methods are different from those obtained with binary methods (Figure 1). Data derived from co-complex studies cannot be directly assigned a binary interpretation. An algorithm or model is needed to translate group-based observations into pairwise interactions. The spoke model is most commonly used, as it produces the minimal number of false positives [12]. An example of networks derived from Y2H versus TAP-MS (Figure 1) illustrates the differences that have to be well understood by any researcher producing or analyzing PPI data.

## The Main Databases and Repositories That Include PPIs

Several previous publications describe databases related to protein interactions [13]–[15]. These reports do not analyze and compare the data sources or the types of interactions that the PPI databases include. Recent debate has questioned how many large-scale or small-scale literature-curated PPI data sets are included in public databases and what is the quality of such data [16]. In this debate, public repositories have stated that their aim is to collect and organize experiments supporting PPIs into comprehensive sets of accurately annotated data, without a biased selection to evidence considered more reliable or otherwise privileged [17]. Regardless, practical users have to know which types of interaction databases are available, what are the differences between them, and which are the most comprehensive and stable repositories.

A comparison of the main databases and repositories that include protein interactions is shown in Table 1, indicating the sources of the data (“PPI Sources”), the types of molecular interactions (“Type of MI”) and the total number of proteins and interactions (where available). Examination of the information in Table 1 defines three different approaches in the collection and presentation of interaction data: (i) primary databases, which include experimentally proven protein interactions coming from either small-scale (Ssc) or large-scale (Lsc) published studies that have been manually curated; (ii) meta-databases, which include only experimentally proven PPIs obtained by consistent integration of several primary databases (sometimes including small sets of original PPI data); (iii) prediction databases, which include mainly predicted PPIs derived using different approaches, combined with experimentally proven PPIs. Computational methods for predicting protein interaction partners were previously reviewed in [18].

**Table 1:** Description of PPI databases and repositories.

Acronym	Database Full Name and URL	PPI Sources	Type of MI	Species	*n* Proteins (Dec. 2009)	*n* Interactions (Dec. 2009)
**Primary Databases: PPI experimental data (curated from specific SSc & LSc published studies)**						
**BIND**	Biomolecular Interaction Network Database, http://bond.unleashedinformatics.com/	Ssc & Lsc published studies (literature-curated)	PPIs & others	All	[31,972]	[58,266]
**BioGRID**	Biological General Repository for Interaction Datasets, http://www.thebiogrid.org/	Ssc & Lsc published studies (literature-curated)	PPIs & others	All	[28,717]	[108,691]
**DIP**	Database of Interacting Proteins, http://dip.doe-mbi.ucla.edu/dip/	Ssc & Lsc published studies (literature-curated)	Only PPIs	All	20,728	57,683
**HPRD**	Human Protein Reference Database, http://www.hprd.org/	Ssc & Lsc published studies (literature-curated)	Only PPIs	Human	27,081	38,806
**IntAct**	IntAct Molecular Interaction Database, http://www.ebi.ac.uk/intact/	Ssc & Lsc published studies (literature-curated)	PPIs & others	All	[60,504]	[202,826]
**MINT**	Molecular INTeraction database, http://mint.bio.uniroma2.it/mint/	Ssc & Lsc published studies (literature-curated)	Only PPIs	All	30,089	83,744
**MIPS-MPact**	MIPS protein interaction resource on yeast, http://mips.gsf.de/genre/proj/mpact/	Derived from CYGD	Only PPIs	Yeast	1,500	4,300
**MIPS-MPPI**	MIPS Mammalian Protein-Protein Interaction Database, http://mips.gsf.de/proj/ppi	Ssc published studies (literature-curated)	Only PPIs	Mammalian	982	937
**Meta-Databases: PPI experimental data (integrated and unified from different public repositories)**						
**APID**	Agile Protein Interaction DataAnalyzer, http://bioinfow.dep.usal.es/apid/	BIND, BioGRID, DIP, HPRD, IntAct, MINT	Only PPIs	All	56,460	322,579
**MPIDB**	The Microbial Protein Interaction Database, http://www.jcvi.org/mpidb/	BIND, DIP, IntAct, MINT, other sets (exp & lit.-curated)	Only PPIs	Microbial	7,810	24,295
**PINA**	Protein Interaction Network Analysis platform, http://csbi.ltdk.helsinki.fi/pina/	BioGRID, DIP, HPRD, IntAct, MINT, MPact	Only PPIs	All	[?]	188,823
**Prediction Databases: PPI experimental and predicted data (“functional interactions”, i.e., interactions ** ***lato sensu*** ** derived from different types of data)**						
**MiMI**	Michigan Molecular Interactions, http://mimi.ncibi.org/MimiWeb/	BIND, BioGRID, DIP, HPRD, IntAct, & nonPPI data	PPIs & others	All	[45,452]	[391,386]
**PIPs**	Human PPI Prediction database, http://www.compbio.dundee.ac.uk/www-pips/	BIND, DIP, HPRD, OPHID, & nonPPI data	PPIs & others	Human	[?]	[37,606]
**OPHID**	Online Predicted Human Interaction Database, http://ophid.utoronto.ca/	BIND, BioGRID, HPRD, IntAct, MINT, MPact, & nonPPI data	PPIs & others	Human	[?]	[424,066]
**STRING**	Known and Predicted Protein-Protein Interactions, http://string.embl.de/	BIND, BioGRID, DIP, HPRD, IntAct, MINT, & nonPPI data	PPIs & others	All	[2,590,259]	[88,633,860]
**UniHI**	Unified Human Interactome, http://www.mdc-berlin.de/unihi/	BIND, BioGRID, DIP, HPRD, IntAct, MINT, & nonPPI data	PPIs & others	Human	[22,307]	[200,473]

The table divided in three sections: **primary databases**, which include PPIs from large- and small-scale (Lsc & Ssc) experimental data that are usually obtained from curation of research articles (8 resources included: BIND, BioGRID, DIP, HPRD, IntAct, MINT, MIPS-MPACT, MIPS-MPPI); **meta-databases**, which include PPIs derived from integration and unification of several primary repositories (3 resources: APID, MPIDB, PINA); **prediction databases**, which include PPIs from experimental analyses together with predicted PPIs obtained from the analyses of heterogenous biological data (5 resources: MiMI, PIPs, OPHID, STRING, UniHI). The table shows the total number of proteins and interactions that were reported by each repository in December 2009 (as far as we could see in the respective Web site). The numbers are in brackets [ ] when the repository includes PPIs and other types of interactions (e.g., protein-ligand interactions or for the case of prediction databases nonPPI data). The question mark [?] indicates that the number of distinct proteins included is such repository could not be found in the Web.

There is a strong need to distinguish between “experimental” PPIs and “predicted” PPIs in order to avoid misinterpretation of the results provided by one or the other approach. Both types of data can be useful, but it is not the same to test an interaction between protein A and B by Y2H as it is to infer a possible interaction between protein A and B based on their gene co-expression profile. In the first situation, the PPI is experimentally proven, while in the second the PPI is predicted from experimental data obtained for the corresponding genes, which does not prove a direct protein interaction.

Some of the primary databases are DIP [19], IntAct [20], and MINT [21], which are the core founders of IMEx, the international consortium of molecular interaction (MI) database providers. This consortium, together with HUPO Proteomics Standards Initiative (PSI) (http://www.psidev.info/), has defined the standard MIMIx (minimal information about a molecular interaction) [22], which is proposed to improve data quality and curation of MIs. Regarding meta-databases, APID [23], [24] and PINA [25] represent to date the most comprehensive efforts to integrate PPI experimental data in single platforms.

## Analysis of Coverage and Ways to Improve PPI Reliability

There are clear discrepancies in current estimations of the real size of the protein interactomes, even for the well-studied unicellular model organism *Saccharomyces cerevisiae*. An empirical estimate of the complete binary protein interactome in *S. cerevisiae* [9] finds ∼18,000±4,500 PPIs, which is consistent with a previous computational estimate of 16,000 to 26,000 interactions [26]. Others estimate more than 30,000 potential interactions between the ∼6,000 proteins of this yeast [4], and some databases with only experimental data currently list more than 50,000 binary interactions between yeast proteins. These observations indicate that some of the experimentally determined PPIs included in the databases are most probably false positives, and therefore ways are needed to obtain more reliable PPIs by estimating the error rates in the data.

**Figure 2:**
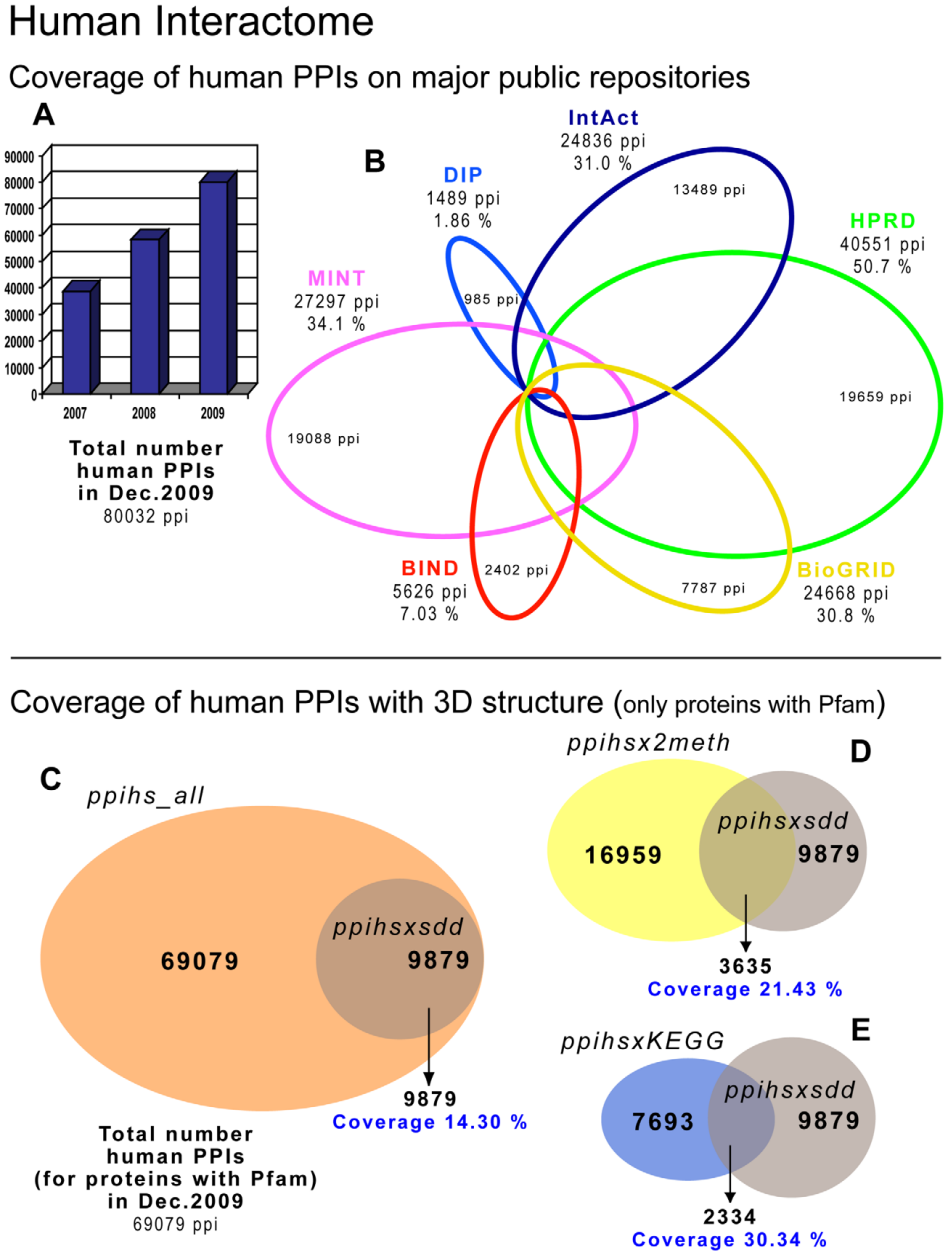
Human interactome: overlap of six databases and coverage of 3-D structural data. Analysis of human interactome PPI data showing the coverage of six major primary databases (BIND, BioGRID, DIP, HPRD, IntAct, and MINT), according to the integration provided by the meta-database APID. (**A**) Growth of the total number of human PPIs during the last 3 years. (**B**) Number of PPIs obtained from each primary repository showing the % (with respect to the total number of PPIs: 80,032 in December 2009) and the number of PPIs only reported by each database (shown inside the corresponding sector of the Venn diagram). Coverage and intersection of PPIs with 3-D structural information: (**C**) Intersection between the PPIs of all human proteins that have at least one *Pfam* annotated (69,079 interactions, called *ppihs_all*) and the PPIs that include proteins with 3-D structural information (9,879 interactions, called *ppihsxsdd*); (**D**) intersection between the PPIs with 3-D structural information and a more stringent interactome constituted by PPIs proven at least by two experimental methods (16,959 interactions, called *ppihsx2meth*); (**E**) intersection between the PPIs with 3-D structural information and more stringent interactome constituted by interactions between proteins that are annotated to the same KEGG functional pathway (7,693 interactions, called *ppihsxKEGG*).

A first obstacle to evaluate the reliability of PPIs is the low coverage of the databases for each specific interactome. One way to increase coverage is to integrate data reported by different primary databases. Each database lacks a substantial proportion of the total reported PPIs [15], [23]. For example, the data on human PPIs coming from six different primary databases show a small overlap (Figure 2) (using a total of 80,032 interactions included in APID in December 2009). In fact, there are only three PPIs that are actually contained in all six of these resources (i.e., full overlap). The number of PPIs exclusively reported by each database is large (as indicated inside the corresponding colored circle of the Venn diagram in Figure 2). The graph in Figure 2A shows the observed growth of human PPIs in the past 3 years. HPRD and MINT are the primary databases that include the most human PPIs: 50.7% and 34.1%, respectively.

Once the coverage is the best possible for a given interactome, strategies for selecting reliable PPIs are needed. A possible solution is to incorporate 3-D structural information about the interacting proteins. This is based on the principle that direct physical PPIs occur via specific structural interfaces, which can often be associated to domain pairs of known 3-D structure, i.e., to structural domain–domain interactions (sddis). Integration of sddi data with PPI data may help to reduce false positives and can be used to validate large-scale protein interaction data [27].

To show the coverage of 3-D structural data on the known human protein–protein interactome, we produced three different subsets of this interactome at three levels of confidence: (i) a subset of the complete human PPI data including only the proteins that have at least one *Pfam* domain assigned: 69,079 interactions, called *ppihs_all* (Figure 2C); (ii) a subset of *ppihs_all* with only the interactions that have been validated by at least two experimental methods that demonstrate the interaction or by the same experimental method reported in at least two independently published articles: 16,959 interactions, called *ppihsx2meth* (Figure 2D); (iii) a subset of *ppihs_all* with only the interactions corresponding to proteins that work together in the same KEGG biological pathway: 7,693 interactions, called *ppihsxKEGG* (Figure 2E) (http://www.genome.jp/kegg/pathway.html). Besides these three groups, we built another subset including all protein pairs supported by structural domain–domain interactions (called *ppihsxsdd*), selecting human PPIs that had at least one structural domain pair reported by one sddi resource. The sddi repositories are based on the analysis of 3-D structural interactions between protein domains taken from the PDB database [27]. The *ppihsxsdd* subset includes 3,688 human proteins and 9,879 interactions. The Venn diagrams (Figure 2C–2E) indicate that the coverage of structural data increases from 14.3% to 21.4% and 30.3%, following the increase in “stringency” of the interactome datasets. Therefore, the structural validation can help to increase reliability of PPI data, as shown by the larger percentage (21.4%) of sddis getting included in the interactome proven by two methods (*ppihsx2meth*).

## Networks Derived from PPIs Compared to Canonical Pathways

In several PPI repositories, it is a straightforward process to obtain all the proteins that interact with a given query protein and from those to build a corresponding network of molecular interactions. Several bioinformatic tools have been developed to represent and explore such PPI networks. Probably the most useful ones are associated with Cytoscape (http://www.cytoscape.org/), an open-source bioinformatics software platform for visualizing molecular interaction networks and biological pathways and for integrating these networks with annotations and other types of data [28], [29]. There are several Cytoscape plug-ins that can be used to download and explore PPIs: APID2NET allows direct data import from the APID repository [24]; BiogridPlugin allows import from BioGRID [30]; MiMIplugin retrieves molecular interactions from the MiMI database [31]; and IntActWSClient, StringWSClient, and PathwayCommons WSC are Web service clients accessible from Cytoscape through the Web Service Client Manager that provide connectivity to IntAct, STRING [20], [32], or Pathway Commons (http://www.pathwaycommons.org/).

**Figure 3:**
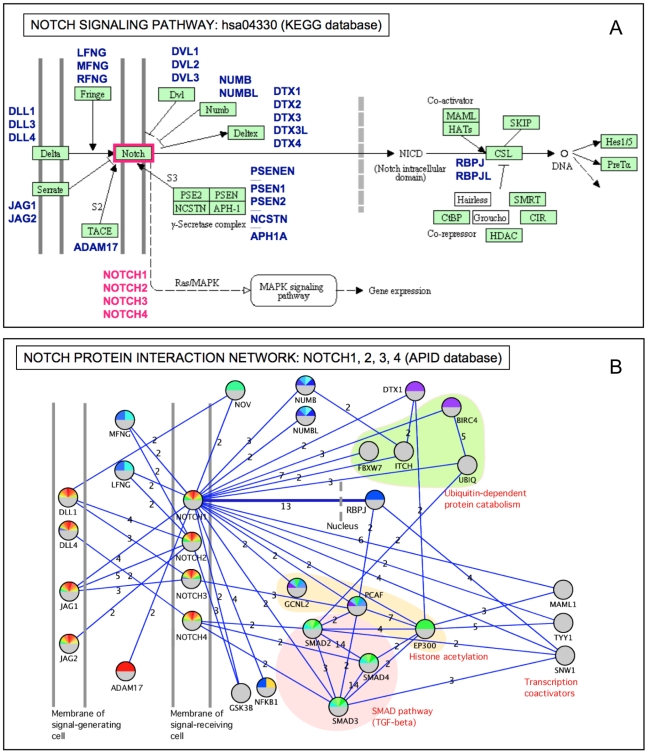
A network derived from PPIs compared to the related canonical pathway. Comparison between a known pathway (NOTCH signaling pathway, taken from the KEGG database, ID: hsa04330) and the corresponding interactome network build using the proteins that interact with human NOTCH proteins. The top panel (**A**) shows the pathway including nine proteins (green boxes) directly connected to NOTCH. In this pathway, the central element is the NOTCH receptor and the interaction of its intracellular domain (called NICD) with protein RBPJ. The bottom panel (**B**) shows the NOTCH PPI network (built with Cytoscape and APID2NET), including all interactors proven with at least two different experiments. The number of experiments is indicated next to each link (blue line). The PPI network provides complementry information to the KEGG pathway, revealing the particular links of each of the four NOTCH paralogous proteins (NOTCH1, 2, 3, and 4) present in the human proteome. The biomolecular elements included in both networks are quite similar and the information that can be deduced from them is complementary. This can be seen in the interaction between NOTCH and RBPJ that drives the central signaling of the pathway and it is present in both networks.

It is worthwhile to compare the characteristics and information provided by a PPI network with the information about the corresponding canonical pathway involving the same proteins. We present a practical example by comparing the human NOTCH signaling pathway to the corresponding PPI network obtained with the interactions of the four NOTCH human proteins (Figure 3). The first one was directly taken from KEGG (ID: hsa04330) (Figure 3A), which is probably the most complete, well-integrated, and annotated database of biological pathways [33], [34]. The second network was built using APID2NET and Cytoscape, retrieving the proteins that interact with NOTCH1, NOTCH2, NOTCH3, or NOTCH4 (UniProt IDs: P46531, Q04721, Q9UM47, Q99466) in interactions demonstrated by at least two different experiments (Figure 3B).

The KEGG pathway representation does not distinguish the relations between the four NOTCH paralogous proteins, while the PPI network separates the links proven for each NOTCH paralogous protein. By contrast, the KEGG pathway representation distinguishes the direction and properties of the links, while the PPI network does not include such directional information. The biomolecular elements (i.e., the nodes) in both networks are generally similar, and the information that can be deduced from them is complementary, each single view being enriched by the other. The γ-secretase complex is not included in the PPI network, while the interaction of NOTCH with the SMAD pathway is not present in the KEGG network. The central role of NOTCH and RBPJ is represented in both views (Figure 3A and 3B), showing that this intracellular interaction drives the signaling pathway. In conclusion, the use of PPI data combined with related pathways allows for a useful and detailed exploration of protein networks. This approach may bring about better comprehension of the complex functional roles that the proteins play by physically interacting in living systems.

## Summary and Guidance for Learning More

This tutorial presents an up to date overview of PPIs, which are defined as *specific physical contacts between protein pairs that occur by selective molecular docking in a particular biological context*. Following this definition, we present some concepts related to the experimental methods used to determine PPIs, the types of biological repositories that include PPIs, and some strategies for analyzing the quality of protein interactions. Adequate description of the main characteristics of each PPI, including complete biological information about the proteins, is essential for building reliable protein interaction networks. As a guide for building and analyzing interactome networks, the tutorial provides a broad collection of references about PPI data resources [19]–[21], [23], [25], [30]–[32] and about related bioinformatic tools [24], [28]–[32]. PPI networks can provide a complementary view to the biological pathways that enclose the corresponding proteins. Looking forward, two main challenges remain for the field and for database providers: (i) a better filtering of false positives in PPI collections and (ii) an adequate distinction of the biological context that specifies and determines the existence or not of a given PPI at a given biological situation.
